# Supplementary figures and images for: Cell-Culture Adaptation of H3N2 Influenza Virus Impacts Acid Stability and Reduces Airborne Transmission in Ferret Model
Source: Viruses. 2021 Apr 21;13(5):719. doi: 10.3390/v13050719 (PMC8143181; doi:10.3390/v13050719)

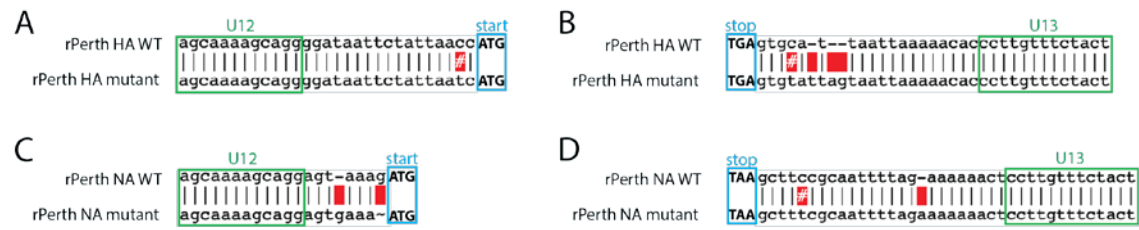

Supplementary File 1

Supplement: Supplementary file 1 [file viruses-13-00719-s001.zip › viruses-1157803-supplementary.pdf]
